# A Review of the Action of Magnesium on Several Processes Involved in the Modulation of Hematopoiesis

**DOI:** 10.3390/ijms21197084

**Published:** 2020-09-25

**Authors:** Fabiana da Silva Lima, Ricardo Ambrósio Fock

**Affiliations:** 1 Department of Food and Experimental Nutrition, School of Pharmaceutical Sciences, University of São Paulo, São Paulo 05508-000, Brazil; fabianadsl@usp.br; 2 Laboratory of Experimental Hematology, Department of Clinical and Toxicological Analysis, School of Pharmaceutical Sciences, University of São Paulo, Avenida Lineu Prestes, 580-Bloco 17, São Paulo 05508-000, Brazil

**Keywords:** magnesium, hematopoietic tissue, hematopoietic cells, bone marrow, immune cells

## Abstract

Magnesium (Mg^2+^) is an essential mineral for the functioning and maintenance of the body. Disturbances in Mg^2+^ intracellular homeostasis result in cell-membrane modification, an increase in oxidative stress, alteration in the proliferation mechanism, differentiation, and apoptosis. Mg^2+^ deficiency often results in inflammation, with activation of inflammatory pathways and increased production of proinflammatory cytokines by immune cells. Immune cells and others that make up the blood system are from hematopoietic tissue in the bone marrow. The hematopoietic tissue is a tissue with high indices of renovation, and Mg^2+^ has a pivotal role in the cell replication process, as well as DNA and RNA synthesis. However, the impact of the intra- and extracellular disturbance of Mg^2+^ homeostasis on the hematopoietic tissue is little explored. This review deals specifically with the physiological requirements of Mg^2+^ on hematopoiesis, showing various studies related to the physiological requirements and the effects of deficiency or excess of this mineral on the hematopoiesis regulation, as well as on the specific process of erythropoiesis, granulopoiesis, lymphopoiesis, and thrombopoiesis. The literature selected includes studies in vitro, in animal models, and in humans, giving details about the impact that alterations of Mg^2+^ homeostasis can have on hematopoietic cells and hematopoietic tissue.

## 1. Introduction

In recent years, researches have shown that alterations in Mg^2+^ homeostasis, a result of the inadequacy of consumption or the moderate or severe deficiency of this mineral, in animal or in vitro and human models, can result in inflammation. Mg^2+^ deficiency frequently results in nuclear factor kappa B (NF-ĸB) pathway activation in immune cells with increased production of proinflammatory cytokines and acute-phase proteins such as interleukin (IL)-6, tumor necrosis factor alpha (TNF-α), and C-reactive protein (CRP), and it is related to the development of chronic diseases [1]. The number of experimental studies that demonstrated how Mg^2+^ deficiency alters the functioning of cells from hematopoietic origin are uncountable. These alterations include increased numbers of leukocytes in the peripheral blood, activation of cells such as neutrophils and macrophages, decreased antibody production, and the arrest of the cell cycle, thereby altering cell-cycle regulation and the activity of cyclins and cyclin-dependent kinases (CDKs) [2,3,4,5,6]. In lymphocytes, data indicate a role for magnesium in cell proliferation, also modulating the development of B cells and immunoglobulin production, as well as affecting functions of T cells [2,7,8]. In this context, much remains to be explored about the importance of intracellular Mg^2+^ homeostasis in cells of hematopoietic origin.

In addition to leukocytes, we and others have shown that Mg^2+^ is important for mechanisms of proliferation, differentiation, and immunomodulation of MSCs (mesenchymal stem cells), and these cells are relevant to both the immune system and the hematopoietic tissue due to its immunomodulatory properties and differentiation capacity in osteoblasts, adipocytes, and chondrocytes [9,10,11]. In vitro studies have shown that reduced Mg^2+^ concentration in the culture cell medium is able to decrease the expression of genes such as *ALP* (alkaline phosphatase)*, COL1* (Collagenase I) and *RUNX*2 (RUNX family transcription factor 2), during the differentiation of bone-marrow-derived MSCs into osteoblasts [12]. Osteoblasts are very important for hematopoietic tissue [13,14]. However, the impact of changes in Mg^2+^ homeostasis in osteoblasts on bone marrow and, consequently, on hematopoietic tissue is unknown.

Studies showing the effect of disturbances in Mg^2+^ homeostasis on the mechanisms of differentiation, proliferation, and maturation of hematopoietic cells in the bone marrow are rare, and there are few references in recent decades about this topic, whereby the majority of the results were obtained only in experimental models [15]. These studies demonstrated the impact of Mg^2+^ deficiency on peripheral blood cells [4,5] or on the lymphocyte maturation process, as well as on thrombopoiesis, with a focus on the transient receptor potential cation channel subfamily M member 7 (TRPM7) channel [16,17]. Several studies raised the importance of intracellular Mg^2+^ homeostasis via the TRPM7 channel, which in addition to being permeable to Mg^2+^, is also permeable to Ca^2+^ and Zn^2+^, relevant for processes such as growth, survival, differentiation, and cell migration [18,19,20,21,22,23]. However, the importance of TRPM7 in Mg^2+^ homeostasis has been questioned, especially in T cells, where TRPM7 deletion did not affect acute uptake or maintenance of total cellular Mg^2+^; this is because, in immune cells, Mg^2+^ may be taken up by magnesium transporter 1 (MagT1) [7]. Nevertheless, these processes are not entirely clear and remain need to be investigated.

Although the impact of alterations in Mg^2+^ homeostasis needs to be clarified, especially Mg^2+^ deficiency in bone marrow and hematopoiesis, many clues indicate that changes in Mg^2+^ concentrations may have profound impacts on hematopoietic tissue. In the last few decades, several studies pointed indirectly to this when they related the importance of Mg^2+^ for cell-cycle progress, cell differentiation, apoptosis, the balance between osteoblast and adipocytes, etc. [3,24,25,26,27,28,29,30]. In light of the above, our objective in this review is to discuss how changes in Mg^2+^ homeostasis could influence bone marrow and, consequently, the hematopoiesis process, and what mechanisms may be involved. Understanding how this micronutrient can influence the hematopoietic process is relevant to highlight the importance of this mineral in the complex physiology of blood cell production, providing insight into the roles of this mineral in the physiological process or even in some hematopoietic pathologies. Given the scarcity of data related to the impact of changes on Mg^2+^ homeostasis in hematopoietic tissue, we focused on publications that evaluated the importance of Mg^2+^ for various types of cells of hematopoietic origin and stromal cells, with the latter being fundamental for the maintenance of hematopoietic tissue. The main findings of this review are compiled in Table 1.

It is important to note that the literature has many mechanistic studies performed in vitro using hematopoietic or lineage cells cultivated in combination with a supraphysiological concentration of Mg^2+^ or even using reduced or absent Mg^2+^ concentrations, which is often incompatible with real life. Therefore, understanding how changes in Mg^2+^ homeostasis can influence hematopoietic cells in vivo is a challenge. Since, the serum Mg^2+^ does not reflect its intracellular content, serum Mg^2+^ levels below the reference range (commonly used to diagnose deficiency) reflect only severe deficiency of this mineral, whereas the effects of moderate deficiency may be discrete and clinically underestimated.

## 2. The Role of Mg^2+^ in Hematopoiesis

### 2.1. The Hematopoietic Microenvironment

Since the 1920s, a series of studies made it possible to understand the role of hematopoietic stem cells (HSCs) and hematopoiesis [57,58,59]. Together, several studies showed that hematopoiesis is a complex, dynamic, and continuous process, in which HSCs proliferate, differentiate, and aggregate in the myeloid or lymphoid lineages, giving rise to the different types of cells that make up the blood system [60,61,62,63]. These processes occur in the bone marrow of mammalian adults under normal conditions and are highly regulated such that the hematopoietic system supplies thousands of mature blood cells to the body daily. The hematopoietic cells perform many essential functions for the survival of the organism, such as oxygen supply, regulation of blood homeostasis, and control of adaptive and innate immunity [64,65]. In this way, the continuous production of many blood cell types requires a highly regulated yet highly responsive system. In 1978, one of the first reports was published that proposed a concept called “niche” in the bone marrow microenvironment, which is a complex multicellular network that provides molecular cues and physical interactions that are essential for the proliferation, self-renewal, differentiation, and migration of HSCs and progenitor cells [66].

The cellular constituents of the bone marrow microenvironment largely derive from a common progenitor of mesenchymal origin called mesenchymal stem cells (MSCs); these cells are rare hematopoiesis-supporting stromal cells that have self-renewal potential and the capacity to differentiate into bone, fat, and cartilage. Furthermore, it is postulated that MSCs are essential in the formation and control of hematopoietic niches, which include the osteoblastic, vascular, and perivascular niches [67]. These niches can modulate different aspects of the hematopoiesis process, having the important role of cell–cell interaction and contact for the medullary microenvironment, as well as the production of soluble factors (cytokines and growth factors) and formation of an extracellular matrix that forms the supporting parenchyma [66,68]. All these elements act in different ways for the homeostasis of HSCs, as well as for the differentiation process of HSCs and progenitor hematopoietic cells and the mobilization of mature cells into peripheral blood [69,70,71]. However, the initiating factors that determine the differentiation process of HSCs are not entirely clear, and it is unknown how changes in Mg^2+^ homeostasis can influence the bone marrow microenvironment. However, on the basis of studies that demonstrated the influence of this mineral on the cells that make up the medullary microenvironment, which are cells that support and maintain hematopoiesis, it is possible to infer that Mg^2+^ is very important for the homeostasis of this tissue, since it is an essential mineral for several cellular functions.

#### 2.1.1. The Influence of Mg^2+^ in Mesenchymal Stem Cells, Osteoblasts, and Adipocytes

Among the cells that make up the stroma, bone marrow mesenchymal stem cells (BMSCs) play a central role in the bone marrow microenvironment due to their ability to differentiate into strains that are very important for the maintenance of hematopoietic tissue, such as osteoblasts and adipocytes. A balance in the differentiation between osteoblasts and adipocytes is extremely significant because adipocytes are a negative regulator of hematopoiesis and osteoblasts are promoters [14,72,73]. Alterations in Mg^2+^ homeostasis are implicated in changes in the differentiation process of MSCs; whereas their increase promotes osteoblastogenesis, Mg^2+^ deficiency appears to be detrimental to bone health [74,75]. In BMSCs from experimental models, an increase in Mg^2+^ concentration elevated the expression of *RUNX2, OSTERIX*, and *OSTEOCALCIN* genes, increased the levels of cyclin D1 and proliferating cell nuclear antigen (PCNA) proteins, and induced the activation of Notch signaling [31]. Studies with osteoblasts from animal models verified that 6 mM and 10 mM Mg^2+^ upregulated *RUNX2, BMP2, ALP, OPN*, and *COL1* expression and increased the phosphoinositide 3-kinase (PI3K)/protein kinase B (Akt) signaling pathway [32]. In addition to *RUNX2* and *ALP*, researchers showed in hFOB1.19 human osteoblast cells that increased of Mg^2+^ ions might also enhance osteoblastogenesis across the TRPM7/PI3K signaling pathway, as PI3K phosphorylation occurs via TRPM7 and leads to the migration of osteoblasts, whereas knockdown of *TRPM7* gene decreased alkaline phosphatase (ALP) activity [33].

Human mesenchymal stem cell (hMSC) studies demonstrated that TRPM7 and MagT1 may be important for osteoblastogenesis due to the increase in their expression during differentiation into osteoblasts. Moreover, downregulation of TRPM7 and MagT1 leads to autophagy, which could lead to accelerating osteoblastic differentiation, leading the authors to describe TRPM7 and MagT1 as possible osteoblastogenesis regulators [76,77]. However, others also showed that higher Mg^2+^ may have adverse effects on bone metabolism, maybe in part due to changes in the expression of TRPM7 and in the homeostasis of other important metal ions for bone tissue, in addition to inhibiting ALP activity in osteoblasts [34].

In experimental models, dietary Mg^2+^ deficiency leads to decreased bone mineral content in the trabecular compartment, decreased osteoblast and increased osteoclast numbers, reduced alkaline phosphatase (ALP) and osteocalcin, and elevated TNF-α, substance P, and IL-1 observed in the bone marrow microenvironment following immunohistochemical analysis in the bone [35,78]. In addition, observed changes were seen in the receptor activator of nuclear factor κB ligand (RANKL) and osteoprotegerin (OPG) rates in the tibia of animals with Mg^2+^ deficiency, suggesting greater stimulus for bone resorption [79].

In view of these studies from human and experimental models, Mg^2+^ deficiency can affect bone health and is related to low BMD and bone mass, where the latter are conditions that can trigger an increase of adipogenesis, leading to an imbalance of osteoblasts/adipocytes in bone marrow [80,81,82,83]. In this way, Mg^2+^ deficiency could indirectly affect the osteoblast/adipocyte taxa. An in vitro study with cell line C3H10T1/2 verified that the increase in H_2_O_2_, a reactive oxygen species (ROS), resulted in higher adipogenesis, decreased sirtuin-1 (SIRT1) protein expression, increased expression levels of Kruppel-like factor 5 (KLF5) and peroxisome proliferator-activated receptor (PPAR) γ2, and decreased RUNX2 levels, resulting in enhancing adipogenesis to the detriment to osteogenesis [84]. Mg^2+^ deficiency is also a condition that leads to oxidative stress, increasing H_2_O_2_ and lipid peroxidation [85,86,87]; therefore, Mg^2+^ deficiency could affect the osteoblast/adipocyte balance not only by increasing adipogenesis due to alterations in bone homeostasis, but also by raising oxidative stress, elevating inducible nitric oxide synthase (iNOS) and H_2_O_2_ [84,88,89]. Our group and others also observed previously that nutritional aspects are extremely relevant for the osteoblast/adipocyte balance [90,91]. It is worth mentioning that, although some studies showed that bone marrow adipocytes may have a relevant role in hematopoiesis [91,92], an increase in these cells in bone marrow is seen as a negative hematopoiesis regulator [72] and is related to several diseases [81,93,94].

#### 2.1.2. Mg^2+^ and Endothelial Cells

The microarchitecture of the medullary microenvironment is composed of different types of vessels (arterial and sinusoidal vessels, arterioles, and capillaries), resulting in an upper vascularized network composed of endothelial cells (ECs), which are indispensable for modulating HSCs [95,96]. ECs in the bone marrow microenvironment are a crucial component for niche vascular homeostasis, especially for their role in sustaining HSCs [97]. Stem-cell factor (SCF) is important for the maintenance of HSCs, and it was reported that SCF inhibition from ECs using Tie2-cre leads to exhausting HSCs in bone marrow, reinforcing the significance of ECs for the support of HSCs [98]. Moreover, ECs also support self-renewal and expansion of HSCs with the involvement of Notch signaling [99,100].

The sinusoidal and arterial niches seem to influence the balance between proliferation and quiescence of HSCs. Many studies found that the permeability properties of vessels play a central role in this balance. Arterial vessels with less permeability are capable of maintaining HSCs in a resting state. On the other hand, higher-permeability sinusoids promote HSCs and progenitor cells and, therefore, alterations in these microenvironments may be related to hematological abnormalities [96,101,102]. It is unknown how Mg^2+^ influences endothelial cells from bone marrow. However, Mg^2+^ deficiency is a condition that triggers endothelial dysfunction [103]. In vitro, murine microvascular endothelial 1G11 cells cultured with lower Mg^2+^ resulted in less proliferation, altered migration, and increased IL-6, NOS activity, and vascular cell adhesion molecule (VCAM) expression [36], whereas increased levels of plasminogen activator inhibitor (PAI-1) and IL-1 were also observed during Mg^2+^ deficiency [104].

**Figure 1:**
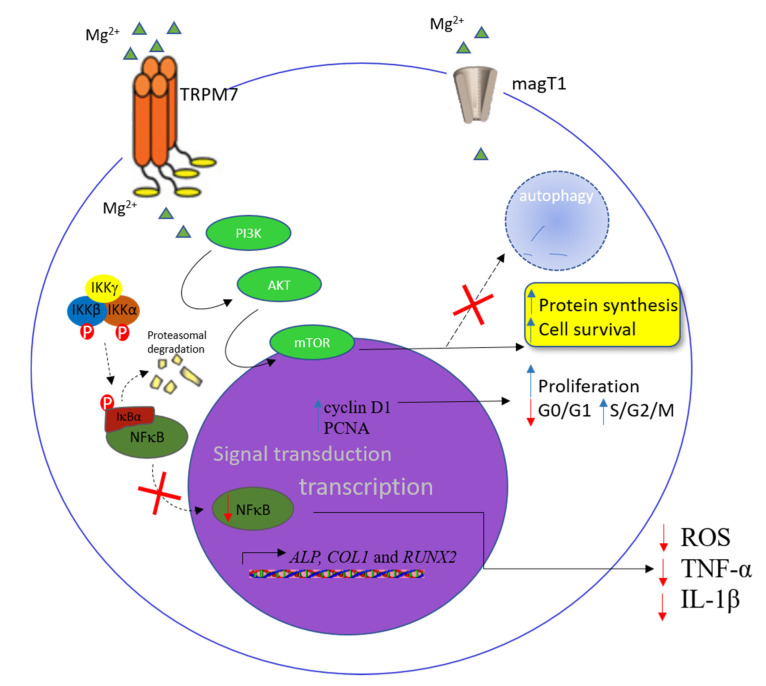
On the hematopoietic cell surface, transient receptor potential cation channel subfamily M member 7 (TRPM7) and magnesium transporter 1(MagT1) facilitate magnesium influx into the cell. In the cytoplasm, the phosphoinositide 3-kinase (PI3K)/protein kinase B (Akt)/mammalian target of rapamycin (mTOR) pathway is magnesium-sensitive, and the activation of this signaling cascade induces cell growth, proliferation, and the inhibition of autophagy. Additionally, increased Mg^2+^ concentration is able to decrease proinflammatory cytokine production, by inhibiting the nuclear translocation and phosphorylation of nuclear factor kappa B (NF-κB) and rise in basal inhibitor of NF-κB (IĸBα) levels, downregulating tumor necrosis factor alpha (TNF-α) and interleukin (IL)-1β release, and reducing ROS production. Solid black arrows indicate the pathway activated, while dot black arrows indicate the pathways inhibited. Red arrows pointing down indicates reduction, while blue arrows pointing up indicates increase.

Other studies, using different sources of endothelial cells, also showed interesting results. In human umbilical vein endothelial cells (HUVECs), Mg^2+^ deficiency triggered higher NF-ĸB activation, along with increases in IL-8, regulated upon activation, normal T cell expressed and secreted (RANTES), and granulocyte macrophage colony-stimulating factor (GM-CSF) [37]. The TRPM7 channel is permeable to Mg^2+^ and relevant for intracellular homeostasis of this mineral, and it appears to play an important role in endothelial function [105]. Human microvascular endothelial cells (HMECs) cultured with a lower concentration of Mg^2+^ resulted in impaired proliferation, increasing the number of cells in the gap (G_0_–G_1_) phase of the cell cycle and downregulation of TRPM7, whereas silencing of TRPM7 showed similar results in this model [38]. In an experimental model, TRPM7 and MagT1 were seen as relevant for Mg^2+^ homeostasis in endothelial cells [39]. In this same study, the deficiency of Mg^2+^ increased the levels of reactive oxygen species (ROS), prostaglandin E2 (PGE2), TNF-α, and IL-1β, and altered the messenger RNA (mRNA) expression of *ICAM-1* and *VCAM-1*. In addition, less viability and cell proliferation were observed during Mg^2+^ deficiency, along with changes in endothelial permeability, and a possible role in regulating the integrity of the endothelial barrier through the sphingosine-1-phosphate receptor 1 (S1P1)–Ras-related C3 botulinum toxin substrate 1 (Rac1) pathway. On the other hand, the authors found that treatment with Mg^2+^ was able to reestablish endothelial homeostasis [39] (Figure 1).

In humans, the influence of Mg^2+^ in endothelial function is controversial. On the one hand, Mg^2+^ supplementation appears to be beneficial for endothelial function. A randomized, double-blind clinical trial with hypertensive women showed that Mg^2+^ supplementation improved endothelial function [40]. A meta-analysis evaluated the effects of Mg^2+^ supplementation on endothelial function and found that treatment with Mg^2+^ improves the flow-mediated dilation (FMD) parameter [106]. On the other hand, a study examined the effects of Mg^2+^ supplementation on the endothelial function of overweight and obese individuals, middle-aged adults, and the elderly, subjected to a daily magnesium supplement of 350 mg for 24 weeks. There was no observed improvement in soluble vascular cell adhesion molecule (sVCAM)-1, soluble intercellular adhesion molecule (sICAM)-1, and soluble endothelial selectin (sE-selectin), along with no change in FMD markers of endothelial function or inflammatory parameters [41].

Taken together, data from the literature show that Mg^2+^ influences endothelial function, partly due to the increase in ROS, activation of the NF-ĸB pathway, and alteration of the expression of ICAM-1 and VCAM-1 during Mg^2+^ deficiency. However, while data from in vitro studies show an improvement in endothelial function with higher Mg^2+^ concentration, in humans, the data are conflicting. It is highly relevant to expand research focused directly on the relationship between changes in Mg^2+^ homeostasis and its impact on endothelial cells that are part of the bone marrow microenvironment, to try to understand how these changes could affect HSC homeostasis and hematopoietic progenitors.

#### 2.1.3. Influence of Mg^2+^ in Macrophages

Bone marrow-resident macrophages are essential for homeostasis of the medullary microenvironment due to its capacity to act in the maintenance of HSC niches and to regulate hematopoiesis [107,108]. Macrophages appear to regulate the retention of HSCs in the bone marrow, altering the expression of genes such as *CXCL12*, *ANGPT1*, *KITL*, and *VCAM1* in Nestin^+^ niche cells from bone marrow, and they are fundamental for normal erythropoiesis [109,110]. Recently, a subset of VCAM-1^+^ macrophages named “usher cells” were demonstrated to be involved in the homing and retention of HSCs and hematopoietic progenitors in a zebrafish model, reinforcing the pivotal role of these cells in the control of bone marrow niches [111]. Furthermore, macrophages are essential for immune regulation, mainly due to their enormous capacity to produce various cytokines, which control both immune response and hematopoiesis [112]. Cytokines are too important for hematopoiesis [113], and the intra- and extracellular concentration of Mg^2+^ can affect their production by leading to the activation of several immune cells including macrophages, but the mechanisms are not entirely clear and the data are conflicting [114]. However, several studies agree that the main initiating factors that contribute to the activation of immune cells and consequently increased inflammatory cytokines during Mg^2+^ deficiency are the increase in intracellular substance P and Ca^2+^ (since Mg^2+^ is a natural blocker of the latter), the rise in oxidative stress, and greater activation of the NF-ĸB pathway [115,116,117].

Additional studies using different sources of macrophages also show an interesting influence of Mg^2+^ levels. In rat alveolar macrophages, Mg^2+^ deficiency in vitro led to an increase of *TNF-α* and *IL-1* mRNA levels with the involvement of Ca^2+^ signaling pathways [42]. In this same study, when these cells were cultured in a control medium or lower Mg^2+^ concentration and stimulated with lipopolysaccharide (LPS), there was a rise in the lower Mg^2+^ condition for both cytokines compared to controls. Yet, Mg^2+^ deficiency is able to increase NO production via iNOS [118]. In order to investigate how different types of liver cells respond to Mg^2+^ deficiency in vitro, RAW264.7 cells grown in Mg^2+^-deficient medium showed increased expression of the *Nqo1* and *Prdx1* genes related to protection against oxidative stress, and this increase could be secondary to the rise in oxidative stress that occurs during Mg^2+^ deficiency [119]. In addition, low Mg^2+^ concentration showed an increase in NF-κB activity in RAW264.7 cells, in addition to alterations in the production and expression of high-mobility group box 1 (HMGB1), a molecule with inflammatory properties [43]. On the other hand, studies showed that increased Mg^2+^ concentration is able to decrease the proinflammatory cytokine production in mononuclear cells from human and animal sources, by inhibiting the nuclear translocation and phosphorylation of NFκB and the rise in basal IĸBα levels [44,120]. Moreover, the effects of preventing inflammation by increased Mg^2+^ concentration in macrophages also include modulation of PI3K/Akt pathway activity, downregulation of TNF-α and IL-1β, enhanced M2 macrophage phenotype, and BMP2 and VEGF expression, with the latter seen in cell subject biomaterials coated with Mg^2+^ [121,122,123] (Figure 1).

Mg^2+^ deficiency results in low-grade chronic inflammation, activating macrophages in tissues and immune cells in peripheral blood, and elevating the concentration of cytokines, such as IL-1, IL-6, and TNF-α [6]. IL-1 and TNF-α are known to induce myelopoiesis, whereas the latter is involved in mechanisms of cell activation and survival in the bone marrow, and both can influence hematopoiesis according to the intensity and duration of their production [124]. Furthermore, it was shown that IL-1 induces PU.1 activation in HSCs with NF-κB pathway involvement, and that chronic exposure to this cytokine triggers changes in HSC homeostasis, principally in the autorenovation mechanisms [125]. In addition, a rise in TNF-α and IL-6 is related to hematopoietic diseases, such as myelodysplasic syndrome and disruption of erythropoiesis [110]; therefore, Mg^2+^ deficiency could negatively affect hematopoiesis. However, it is important to mention that most studies that evaluated the influence of Mg^2+^ in macrophages were performed in vitro, with lineage cells, while many of them were cultured with supraphysiological or the lowest concentrations of Mg^2+^. Thus, it is difficult to conclude how changes in vivo in Mg^2+^ homeostasis may influence human macrophages in the bone marrow, and comprehensive studies on the topic are required.

### 2.2. Mg^2+^ and Erythropoiesis

Erythropoiesis occurs from pluripotent stem cells in the bone marrow, with the erythroid progenitor being BFU-E (erythroid explosion-forming unit), which results in colony-forming units (CFU-E), mature erythroid precursors, reticulocytes, and red blood cells [126]. Erythroid differentiation and maturation occur in erythroblastic islands, and these complex events of proliferation, differentiation, and maturation depend on several factors, such as GATA-binding protein 1 and 2 (GATA 1 and 2), SCF, IGF (insulin-like growth factor), BPA (erythroid burst-promoting activity), IL-3, and EPO (erythropoietin) [127,128,129,130], in addition to nutritional aspects, like iron, folate, and B12 vitamin [131]. Yet, the influence of Mg^2+^ on the erythropoiesis process in the bone marrow is not clear. It is known that there is a relationship between Mg^2+^ and anemia; nevertheless, most studies focused on the importance of this mineral in peripheral blood erythrocytes.

Studies carried out since the 1930s have shown a relationship between Mg^2+^ status and erythrocytes in experimental models. First, it was identified that the Mg^2+^ content in erythrocytes was higher in cases of anemia [132]. Subsequently, a series of studies showed that Mg^2+^ deficiency triggered fetal growth restriction and microcytic anemia, fragmentation of red blood cells, and impaired osmotic fragility in offspring from deficient mothers and in adult rats, while the latter also displayed reticulocytosis, which was attributed to changes in the energy metabolism and membrane of erythrocytes [133,134,135]. Moreover, red blood cells (RBC) from animals with Mg^2+^ deficiency seem to age faster, whereas reticulocytosis indicates that the bone marrow might maintain erythropoiesis during low plasma Mg^2+^ concentration [45]. However, a study showed that Mg^2+^ deficiency increased Fe absorption and concentration in many tissues, but reduced the number of RBCs, possibly due to ineffective erythropoiesis [46]. Macrocytic anemia and decreased exercise capacity were also observed in rats after dietary Mg^2+^ deficiency, in addition to varying the content of K^+^ and Na^+^, raising the activity of cotransport of K–CI, which is related to volume and density in RBC, and decreasing of glutathione in erythrocytes [136,137,138,139].

In humans, the influence of Mg^2+^ status on erythrocytes was also investigated. In preeclampsia, erythrocyte deformability and, consequently, microcirculation are reduced, and Mg^2+^ therapy was able to increase the deformability of these cells [140,141]. A cross-sectional study with adults evaluated the association between consumption of Mg^2+^ and Fe with anemia, and found an inverse association between Mg^2+^ intake and anemia [47]. Researchers analyzed data from the 2009 China Health and Nutrition Survey and verified an association between increased serum Mg^2+^ and decreased risk of anemia in women and men from nine provinces of China. However, this association was significantly greater in women and dependent on ferritin levels [48]. In addition, the dietary pattern (traditional vs. modern pattern) was analyzed in elderly subjects, and a positive association was found between the traditional dietary pattern and anemia with the influence of serum Mg^2+^ [142]. A cross-sectional study with 180 pregnant women (up to 14 weeks of gestation) from Khartoum in Sudan, showed that 65.0% and 57.2% had anemia and Mg^2+^ deficiency, respectively, and that low levels of serum ferritin and Mg^2+^ were associated with anemia [49]. On the other hand, Mg^2+^ supplementation seems to increase the hemoglobin levels and counts of erythrocytes [50].

EPO is a glycoprotein produced mainly by the kidneys and is influenced by hypoxia, with a pivotal role in the mechanisms of survival, proliferation, and differentiation of erythrocytes in the bone marrow [143]. Anemia is a frequent finding in individuals undergoing hemodialysis, mainly due to the decrease in EPO concentrations, but also due to the impaired response to EPO, and an increase in serum Mg^2+^ seems to decrease the risk of a lesser response to EPO [144]. In vitro, lower Mg^2+^ concentrations inhibited the activity of HIF-1α (hypoxia- inducible factor) mediated by ROS through the activation of NF-κB involving T-type calcium channels, with HIFs being an important factor in the promotion of EPO levels [145,146].

Although several studies showed an association between the concentration of Mg^2+^ and the development of anemia and its influence on the erythrocytes in peripheral blood, there is no direct evidence to support how the intra- and extracellular concentration of Mg^2+^ may affect erythropoiesis in the bone marrow. However, we suppose that Mg^2+^ deficiency may partially affect erythropoiesis by altering the NF-κB pathway in macrophages and iron homeostasis, indirectly triggering changes in the membrane and accelerating the aging and destruction of RBCs. Nevertheless, research showing how Mg^2+^ concentration could directly affect erythropoiesis in the bone marrow and how and which pathways are related is important and remains open to investigation. As human studies show a relationship between hypomagnesemia and anemia, it is important to encourage more studies on the topic, given that the correction of hypomagnesemia in addition to Fe could perhaps be considered for the treatment of these hematological alterations.

### 2.3. Mg^2+^ and Granulopoiesis

Among the cells of granulocytic lineage, Mg^2+^ deficiency affects neutrophils most severely, but eosinophils and mast cells can also be affected. Hypomagnesemia triggers a boost of eosinophils in the peripheral blood, increasing the number of mast cells in several tissues and in the bone marrow, impairing their function [51,52,53,147].

Neutrophilia is a common finding during hypomagnesemia in experimental models [4,54,148]. In general, the main justification for this is due to the increased inflammation observed during the deficiency of this mineral. Nevertheless, the mechanisms behind these observations are unclear, and an issue remains if this neutrophilia is a consequence of the inflammatory stimulus resulting from Mg^2+^ deficiency or if molecular changes occur in the development, differentiation, and maturation of this lineage in the bone marrow even before the development of inflammatory phenotype. Perhaps, it is a combination of these two possibilities. However, there is little direct evidence to support which molecular alterations could be involved in the development of neutrophils in the bone marrow during hypomagnesemia. 

Neutrophils are leukocytes that act in the first cell line of defense of organisms against various types of infectious agents, through the mechanism of phagocytosis and production of cytotoxic molecules, and it is not by chance that most bone marrow leukocytes are granulocytic precursors [149]. It is not yet clear, but Mg^2+^ deficiency seems to alter the differentiation and maturation mechanisms of myeloid cells, favoring the granulocytic lineage during the deficiency of this mineral. Alterations in cell-cycle control, intracellular Mg^2+^ levels, and Mg^2+^ compartmentalization may be the main mechanisms behind the neutrophilia observed during hypomagnesemia in vitro [26]. In vivo, an experimental study analyzed the repercussions of long-term consumption of an Mg^2+^-deficient diet on the bone marrow of rats. The authors found that more than half of the bone marrow samples were hypercellular, with a greater number of granulocytic cells, and one animal developed leukemia with granulocytic infiltrate in several organs. Furthermore, cells from granulocytic infiltrate inoculated in newborn animals resulted in leukemia in half of the animals after 3 months of inoculation [15].

The emergence and the steady state of granulopoiesis in the bone marrow are highly regulated, mainly by G-CSF, GM-CSF, CCAAT-enhancer-binding proteins (C/EBPα and C/EBPβ), IL-6, and Janus kinase/signal transducer and activator of transcription (JAK/STAT) pathways [150,151,152]. We conjecture that Mg^2+^ status may alter these signaling pathways, given that changes in granulopoiesis, mainly neutrophilia, occur during hypomagnesemia. Nevertheless, there is no direct evidence of the relationship between Mg^2+^ and these molecular pathways to support this claim, and the repercussions of hypomagnesemia in these signaling pathways and in the granulopoiesis process remain to be elucidated. Additionally, lower Mg^2+^ levels affect the cell-cycle control and aging cell process, beyond upregulation of inflammatory markers, and these changes may affect not only granulopoiesis but also hematopoiesis in general. Yet, it is unknown if Mg^2+^ may affect the C–X–C motif chemokine 12 (CXCL12)/CXCR4 axis that controls the traffic of neutrophils in the bone marrow or whether hypomagnesemia could lead to alterations in dendritic cells [153]. Dendritic cells seem to control the traffic of neutrophils from bone marrow, and their decrease triggers a boost of neutrophils in the peripheral blood, even in the lack of infection [154].

### 2.4. Influence of Mg^2+^ in Lymphopoiesis

Studies showed that extra- and intracellular Mg^2+^ concentrations are important for the activation and proliferation of lymphocytes, and that Mg^2+^ deficiency may affect the function of these cells in peripheral blood, decreasing immunoglobulin production and the number of cells that produce antibodies [2,155,156]. However, the influence of Mg^2+^ homeostasis on the lymphopoiesis process and the progenitor’s lymphoid in bone marrow is less elucidated. Experimental studies showed that severe Mg^2+^ deficiency leads to bone marrow changes that appear to occur at the same time as thymic changes, resulting in hyperplastic bone marrow, lymphoproliferative disorder, and leukemia [157,158]. In addition, dietary Mg^2+^ deficiency resulted in thymic changes, impairing its function, triggering increased necrosis, apoptosis, and phagocytosis, and favoring a tumor microenvironment in rats [86,159,160]. In humans, it was observed that patients with acute lymphocytic leukemia (ALL) have reduced levels of Mg^2+^ in serum and hair; these changes appear to occur in part due to bone alterations resulting from the treatment of the disease [161,162,163,164]. However, the consequences of hypomagnesemia for the health of individuals with hematological diseases and its influence on the development of these diseases are not clear.

The role of TRPM7 and MagT1 in the function and development of lymphocytes is most frequently investigated [165]. Nevertheless, the relationship among intracellular Mg^2+^, TRPM7, and MagT1 homeostasis in the development and function of lymphocytes remains to be established and is not fully understood. In vitro, DT40 cells that lack the *TRPM7* gene showed arrest of the cell cycle in the G_0_–G_1_ phase and led to changes in the PI3K /Akt/mammalian target of rapamycin (mTOR)/S6K pathway, but the increased Mg^2+^ concentration in these cells improved the proliferation levels [166] (Figure 1). A study showed that the absence of the TRPM7 channel in B cells from mice abolished mature B cells in the spleen, lymph nodes, and peripheral blood, and it led to an arrest in the development of pre-pro-B cells and pro-B cells. In addition, it impaired serum levels of immunoglobulins (IgM, IgG, and IgA) and triggered a boost of myeloid cells, especially neutrophils, in the spleen and peripheral blood. In the same study, increased Mg^2+^ concentration in vitro was able to partially improve the development of B cells [167]. However, the same was not noted in T cells, where the deletion of the *TRPM7* gene did not trigger changes in Mg^2+^ homeostasis, while impairing thymopoiesis [7].

One of the most relevant studies about the relationship between lymphocytes and Mg^2+^ was described in the XMEN syndrome (X-linked immunodeficiency Mg^2+^ defect, Epstein–Barr virus infection, and neoplasia), which was first described as Magt1 loss affecting intracellular Mg^2+^ homeostasis and leading to defective T-cell immune responses and uncontrolled EBV infection with increased susceptibility to EBV^+^ lymphoma [168]. This syndrome is a rare primary immunodeficiency caused by hemizygous loss-of-function mutations in the X-linked MagT1 gene in males [169]. Magt1 was initially recognized as an Mg^2+^ transporter, and early studies placed it as a plasma membrane protein; however, more recent data also postulated that Magt1 is localized in the endoplasmic reticulum and is a subunit of the oligosaccharyltransferase (OST) complex and more specifically of the STT3B complex [170]. In this way, the XMEN syndrome and its relationship with Mg^2+^ has now been revised. As the molecular relationship to OST subunits and complexities of Mg^2+^ suggested that pivotal manifestations of XMEN syndrome may rotate around defective glycosylation, in addition to or instead of alterations in Mg^2+^ transport [171]. However, a study from the same group also showed that glycosylation is sensitive to Mg^2+^ levels and that reduced Mg^2+^ impairs immune cell function via the loss of specific glycoproteins [172].

Nevertheless, Mg^2+^ homeostasis is too important for restoring the functions of T cells of individuals that suffer from genetic deficiencies in MagT1 and mutations in the kinase domain of interleukin-2-inducible kinase (ITK). These mutations display impaired function in T cells, such as changes in activation after T-cell receptor (TCR) stimulation, cytotoxicity, and degranulation, resulting in a great predisposition to infections by EBV that is associated with a high risk of development of lymphoma; hence, Mg^2+^ supplementation is important for improving the function of T cells in these conditions [8,173].

### 2.5. Influence of Mg^2+^ in Thrombopoiesis

Regarding thrombopoiesis, the influence of Mg^2+^ in megakaryocytes is not clear. Mg^2+^ is important for platelet homeostasis. Studies showed that lower Mg^2+^ concentrations are involved with platelet-dependent thrombosis and hyperreactivity, whereas increased Mg^2+^ concentration seems to inhibit platelet aggregation, partially altering intracellular Ca^2+^ levels and decreasing the production of molecules involved in this process, such as eicosanoids [174,175,176]. Research found that changes in the intracellular Mg^2+^ concentration in platelets from individuals with obesity and diabetes could be related to the development of future disorders in the coagulation process [177]. A cross-sectional study with Chinese women and men observed that a rise in serum Mg^2+^ level was associated with increased platelet numbers and a lower risk of development of thrombocytopenia [55]. Nevertheless, few studies investigated the role of intra- and extracellular Mg^2+^ concentrations in megakaryocytes in the bone marrow. Mg^2+^ deficiency may promote defects in platelet biogenesis due to changes in the cytoskeleton, promoting changes in platelet function [178]. In an experimental model, dietary Mg^2+^ deficiency impaired megakaryocytes from bone marrow, resulting in a decrease in the number and larger shape than the cells of the control animals [56]. A study showed that disorders in the TRPM7 channel and Mg^2+^ homeostasis in megakaryocytes trigger macrothrombocytopenia by altering the activity of the nonmuscular myosin protein IIA (NMMIIA) and the cytoskeleton, affecting the maturation of platelets in the bone marrow [16].

## 3. Conclusions and Perspectives

Mg^2+^ is the fourth most abundant mineral in the body showing a significant positive correlation with the protein synthesis rate, suggesting a key role of this mineral in the regulation of protein synthesis and in the cell proliferation rate in normal tissue cell populations, especially those with a high turnover such as the hematopoietic system. Due to the importance of this mineral in hematopoiesis, an imbalance can disrupt the hematopoiesis process, as well as the effective activities of mature blood cells. In conclusion, it is crucial to understand the impact of Mg^2+^ on the correct hematopoietic functions, to understanding the mechanisms, and to understand when or how some therapeutic intervention is necessary. However, despite the existence of several studies showing the extensive role of Mg^2+^, more studies are necessary to address the exact impact of this mineral on the hematopoietic system.

## Figures and Tables

**Table 1:** Main findings of relationships between Mg^2+^ and hematopoietic tissue.

Reference	Main Findings	Source
**Mesenchymal Stem Cells and Osteoblasts**		
[31]	Increase in Mg^2+^ enhanced expression of *RUNX2, OSTERIX*, and *OSTEOCALCIN* genes, increased the levels cyclin D1 and PCNA proteins, and induced the activation of Notch signaling.	BMSCs
[32]	Higher Mg^2+^ concentration led to upregulation of RUNX2, BMP2, ALP, OPN, and ColI and increased PI3K/Akt signaling pathway.	Osteoblasts from animal model
[33]	Higher Mg^2+^ levels triggered PI3K phosphorylation via TRPM7 and led to migration of osteoblasts.	hFOB1.19 (human osteoblast cells)
[34]	Increase in Mg^2+^ led to changes in the expression of TRPM7 and in homeostasis of other important metal ions for bone tissue, inhibiting ALP activity in osteoblasts.	SaOS-2 cells and human osteoblasts
[35]	Decreased osteoblast number.	Bone marrow from mice
**Endothelial Cells**		
[36]	Lower Mg^2+^ levels impaired proliferation and migration, and increased IL-6, NOS activity, and VCAM expression.	1G11 cells
[37]	Mg^2+^ deficiency triggered higher NF-ĸB activation, and increased IL-8, RANTES, and GM-CSF.	HUVEC cells
[38]	Lower Mg^2+^ concentration decreased proliferation taxa and downregulation of TRPM7.	HMEC cells
[39]	Mg^2+^ deficiency increased ROS, PGE2, TNFα, and IL-1β levels, triggered changes in mRNA of *ICAM-1* and *VCAM-1*, decreased viability and cell proliferation, triggered changes in the endothelial permeability, and influenced integrity of the endothelial barrier through the S1P1–Rac1 pathway.	ECs from animal model
[40]	Mg^2+^ supplementation improved endothelial function.	Clinical trial
[41]	Daily Mg^2+^ supplement of 350 mg for 24 weeks led to no improvement in soluble vascular cell adhesion molecule (sVCAM)-1, soluble intercellular adhesion molecule (sICAM)-1, and soluble endothelial selectin (sE-selectin), along with no change in FMD markers of endothelial function or inflammatory parameters.	Clinical trial
**Macrophages**		
[42]	Mg^2+^ deficiency increased *TNF-α* and *IL-1* mRNA levels with the involvement of Ca^2+^ signaling pathways.	Rat alveolar macrophages
[43]	Low Mg^2+^ concentration increased NF-κB activity and changed HMGB1 expression.	RAW264.7 cells
[44]	Increased Mg^2+^ concentration inhibited the nuclear translocation and phosphorylation of NF-κB and led to a rise in basal IĸBα levels.	Human PBMCs
**Erythropoiesis**		
[45]	Mg^2+^ deficiency decreased plasma level, and led to faster aging of RBCs, as well as reticulocytosis.	Animal model
[46]	Mg^2+^ deficiency increased Fe absorption and concentration, but reduced the number of RBCs.	Animal model
[47]	Inverse association between Mg^2+^ intake and anemia.	Cross-sectional study
[48]	Association between increased serum Mg^2+^ and decreased risk of anemia in women, dependent on ferritin levels.	Cross-sectional study
[49]	Decreased levels of serum ferritin and Mg^2+^ were associated with anemia in pregnant women.	Cross-sectional study
[50]	Mg^2+^ supplementation raised the hemoglobin levels and counts of erythrocytes.	Clinical trial
**Granulopoiesis**		
[51]	Boost in circulating eosinophils during Mg^2+^ deficiency.	Experimental model
[52]	Mg^2+^ deficiency impaired mast-cell functions.	Experimental model
[53]	Mg^2+^ deficiency increased the mast cells in the bone marrow.	Experimental model
[54]	Increase in the number and activity of PMNs during Mg^2+^ deficiency.	Experimental model
[26]	Lower Mg^2+^ levels triggered granulocytic differentiation and changes in proteins related to cell-cycle control.	HL-60 cells
[15]	Mg^2+^ deficiency resulted in hypercellular bone marrow, as well as a greater number of granulocytic cell; one animal also developed leukemia with granulocytic infiltrate in several organs.	Experimental model
**Thrombopoiesis**		
[55]	Higher serum Mg^2+^ levels were associated with increased platelet numbers and lower risk of development of thrombocytopenia.	Cross-sectional study
[56]	Mg^2+^ deficiency impaired the number and shape of megakaryocytes from bone marrow.	Experimental model
[16]	Changes in the TRPM7 channel and Mg^2+^ homeostasis in megakaryocytes triggered macrothrombocytopenia, altering the activity of the NMMIIA and the cytoskeleton, affecting the maturation of platelets in the bone marrow.	Experimental model

Abbreviations: RUNX2 (RUNX Family Transcription Factor 2); PCNA (Proliferating Cell Nuclear Antigen); BMP2 (Bone Morphogenetic Protein 2); OPN (Osteoprotegerin); ALP (Alkaline Phosphatase), COL1 (Collagenase I); PI3K ( Phosphoinositide 3-Kinase); Akt (Protein Kinase B); TRPM7 (Transient Receptor Potential Cation Channel Subfamily M Member 7); IL-6 (Interleukin-6); NOS (Nitric Oxide Synthase); VCAM (Vascular Cell Adhesion Molecule); NF-ĸB (Nuclear Factor Kappa B); RANTES (Regulated upon activation, normal T cell expressed and secrete); GM-CSF (Granulocyte Macrophage Colony-Stimulating Factor); ROS (Reactive Oxygen Species), PGE2 ( Prostaglandin E2); TNFα (Tumor Necrosis Factor Alpha); ICAM (Intercellular Adhesion Molecule); HGMB1(High-Mobility Group Box 1); IĸBα (Inhibitor of NF-κB); S1P1 (Sphingosine-1-Phosphate Receptor 1 ); Rac1 (Ras-related C3 Botulinum Toxin Substrate 1); RBCs (Red Blood Cells); PMNs (Polymorphonuclear Cells); NMMIIA (Nonmuscular Myosin Protein IIA).

## Abbreviations

1G11	Murine pulmonary vascular endothelial cell
Akt	Protein kinase B
ALL	Acute lymphocytic leukemia
ALP	Alkaline phosphatase
BFU-E	Burst forming unit, erythroid
BMD	Bone mineral density
BMP2	Bone morphogenetic protein 2
C/EBPα	CCAAT-enhancer-binding protein α
C/EBPβ	CCAAT-enhancer-binding protein β
C3H10T1/2	Murine C3H embryo sarcoma
Ca^2+^	Calcium
c-fos	Member of Fos family of transcription factors
CFU-E	Colony-forming unit, erythroid
COL10A1	Collagen type X alpha 1 chain
CXCL12	C–X–C motif chemokine 12
Angpt1	Angiopoietin 1
CXCL12	C–X–C motif chemokine 12
CXCR4	C–X–C chemokine receptor type 4
G-CSF	Granulocyte colony-stimulating factor
DC-STAMP	Dendritic cell-specific transmembrane protein
DNA	Deoxyribonucleic acid
DT40 cell	Chicken B cell line
EBV	Epstein–Barr virus
ECM	Extracellular matrix
EPO	Erythropoietin
Fe	Iron
FMD	Flow-mediated dilation
GATA	GATA-binding protein 1
G-CSF	Granulocyte colony-stimulating factor
GM-CSF	Granulocyte macrophage colony-stimulating factor
H2O2	Hydrogen peroxide
hFOB1.19	Human bone osteoblast; SV40 large T antigen transfected
HIF1-α	Hypoxia-inducible factor 1α
HMGB1	High-mobility group box 1
hMSCs	Human mesenchymal stem cells
HSC	Hematopoietic stem cell
HUVEC	Human umbilical vein endothelial cell
IgA	Immunoglobulin A
IGF2	Insulin-like growth factor 2
IgG	Immunoglobulin G
IgM	Immunoglobulin M
IL-1	Interleukin-1
IL-6	Interleukin-6
IL-8	Interleukin-8
iNOS	Inducible nitric oxide synthase
ITK	Interleukin-2-inducible kinase
JAK/STAT	Janus kinase/signal transducer
K	Potassium
KLF5	Kruppel-like factor 5
MagT1	Magnesium transporter 1
Mg^2+^	Magnesium
mTOR	Mammalian target of rapamycin
Na	Sodium
NF-ĸB	Nuclear factor kappa B
NKG2D	Natural killer group 2 member D
NMMIIA	Nonmuscular myosin protein IIA
OPG	Osteoprotegerin
OST	Oligosaccharyltransferase
PCNA	Proliferating cell nuclear antigen
CRP	C-reactive protein
PGC-1 α	Peroxisome proliferator-activated receptor gamma coactivator 1-alpha
PGE2	Prostaglandin E2
PI3-kinase	Phosphoinositide 3-kinase
PPARγ2	Peroxisome proliferator-activated receptor γ2
PTH	Parathyroid hormone
PU.1	Transcription factor PU.1
Rac1	Ras-related C3 botulinum toxin substrate 1
RANKL	Receptor activator of nuclear factor kappa Β ligand
RANTES	Regulated upon activation, normal T cell expressed and secreted
RBC	Red blood cells
RNA	Ribonucleic acid
ROS	Reactive oxygen species
RUNX2	RUNX family transcription factor 2
S1P1	Sphingosine-1-phosphate receptor 1
S6K	Ribosomal S6 kinase
SaOS-2	Human osteosarcoma cell line
SCF	Stem-cell factor
sE-selectin	Soluble endothelial selectin
sICAM-1	Soluble intercellular adhesion molecule 1
SIRT1	Sirtuin-1
STAT	Signal transducer and activator of transcription
STT3B	Oligosaccharyltransferase complex catalytic subunit B
sVCAM-1	Soluble vascular cell adhesion molecule 1
TCR	T-cell receptor
TNF-α	Tumor necrosis factor α
TRPM7	Transient receptor potential cation channel subfamily M member 7
VCAM1	Vascular cell adhesion molecule 1
VEGF	Vascular endothelial growth factor
